# Prognostic Impact of Obesity, Cardiometabolic Risk Factors, and Vascular Function Markers on Outcomes in Ischemic Cardiomyopathy

**DOI:** 10.3390/jcm14207397

**Published:** 2025-10-20

**Authors:** Konstantinos Mourouzis, Vasiliki Tsigkou, Gerasimos Siasos, Evangelos Oikonomou, Marina Zaromitidou, Evanthia Bletsa, Nikolaos Gouliopoulos, Panagiota K. Stampouloglou, Konstantinos Tsioufis, Manolis Vavuranakis, Dimitris Tousoulis

**Affiliations:** 1 Department of Cardiology, ‘Hippokration’ General Hospital, School of Medicine, National and Kapodistrian University of Athens, 11527 Athens, Greecegsiasos@med.uoa.gr (G.S.); boikono@gmail.com (E.O.); evabletsa@gmail.com (E.B.); ngouliopoulos@yahoo.gr (N.G.); stampoulogloupanagiota@gmail.com (P.K.S.); kptsioufis@gmail.com (K.T.); vavouranakis@gmail.com (M.V.); drtousoulis@hotmail.com (D.T.); 2 Medizinische Klinik und Poliklinik I, LMU Klinikum, Ludwigs-Maximilians-University Munich, 81377 Munich, Germany; 3 Department of Cardiology, ‘Sotiria’ General Hospital, School of Medicine, National and Kapodistrian University of Athens, 11527 Athens, Greece; 4 Cardiovascular Division, Harvard Medical School, Brigham and Women’s Hospital, Boston, MA 02115, USA

**Keywords:** heart failure, ischemic cardiomyopathy, endothelial function, arterial stiffness, flow-mediated dilation, pulse wave velocity, prognosis, ejection fraction, clustering, cardiometabolic risk factors, obesity, obesity paradox

## Abstract

**Background/Objectives**: Ischemic cardiomyopathy is a major cause of morbidity and mortality. Obesity is paradoxically associated with better outcomes, while clustering of cardiometabolic risk factors (CMRFs)—diabetes mellitus, arterial hypertension, and hyperlipidemia—is associated with worse prognosis in heart failure (HF) patients. The interplay between vascular function, obesity and clustering of CMRFs in ischemic HF is not thoroughly investigated. **Methods**: In a prospective, single-center cohort study, 560 patients with ischemic cardiomyopathy were followed for a median of 43 months. Baseline BMI, CMRFs and markers of vascular function including flow-mediated dilation (FMD), and carotid–femoral pulse wave velocity (cf-PWV) were assessed. Major adverse cardiovascular events (MACE), including death, myocardial infarction, coronary revascularization, stroke, and hospitalization for heart failure or other cardiovascular causes, were recorded. Cox proportional hazards models and cubic spline analyses evaluated associations and nonlinear relationships. **Results**: Obesity was independently associated with a 50% lower risk of MACE (HR 0.50; 95% CI 0.32–0.981; *p* = 0.01) and improvement of FMD by 1% corresponded to a 7% reduction in MACE risk (HR 0.93; 95% CI 0.87–0.99; *p* = 0.03) after adjusting for multiple confounders. Clustering of all three CMRFs predicted greater MACE risk (HR 1.42; 95% CI 1.03–1.95; *p* = 0.03). No significant differences in FMD or cf-PWV were observed across BMI groups. cf-PWV values were impaired among patients with all 3 CMRFs but cf-PWV did not predict MACE. **Conclusions**: Higher BMI and FMD each independently predict improved outcomes in ischemic cardiomyopathy. The clustering of cardiometabolic risk factors is a strong predictor of adverse events.

## 1. Introduction

Heart failure (HF) is a complex syndrome characterized by structural and functional cardiac impairment, affecting approximately 56 million people globally, with prevalence rising due to aging and common comorbidities [1]. Coronary artery disease (CAD) may lead to ischemic cardiomyopathy, which is a predominant HF subtype, especially in Western populations [2]. Accurate characterization of HF subtypes—HF with reduced ejection fraction (HFrEF), mildly reduced EF (HFmrEF), and preserved EF (HFpEF)—is essential for guiding personalized treatment strategies [3,4].

Cardiometabolic risk factors (CMRFs) such as diabetes mellitus, hypertension, and dyslipidemia may cluster in HF patients, influencing both vascular function and clinical outcomes [5,6,7]. Conversely, obesity contributes to the manifestation of cardiovascular disease and HF but paradoxically associates with improved HF prognosis, something which in the literature is described as “obesity paradox in HF” [8,9,10]. The mechanisms remain unclear, particularly regarding the roles of endothelial function and arterial stiffness.

Endothelial dysfunction plays a fundamental role in the pathogenesis and progression of cardiovascular diseases, including ischemic HF, by contributing to adverse vascular and cardiac remodeling [11,12]. Evidence shows that patients with HF of ischemic etiology may exhibit worse endothelial function compared to HF of non-ischemic origin [13]. Accumulating data demonstrates that endothelial dysfunction can predict short- and long-term outcomes in patients with heart failure [14,15]. Flow-mediated dilation (FMD) of the brachial artery is a widely accepted, non-invasive marker of endothelial function, reflecting nitric oxide–mediated vasodilation and vascular health. Additionally, arterial stiffness—commonly assessed by carotid–femoral pulse wave velocity (cf-PWV)—correlates with increased cardiovascular risk and poorer HF prognosis, especially when integrated with traditional risk factors [11] although divergent findings regarding its predictive value in ischemic cardiomyopathy also exist [14,16].

The current study evaluated the influence of obesity and clustered CMRF risk factors on FMD and cf-PWV values in patients with ischemic cardiomyopathy. It additionally aimed to investigate the prognostic significance of obesity, the clustering of cardiometabolic risk factors—diabetes mellitus, arterial hypertension, and hyperlipidemia—as well as biomarkers of vascular function, FMD and cf-PWV, for cardiovascular outcomes in patients with ischemic cardiomyopathy.

## 2. Methods

### 2.1. Study Population

This was a single-center, prospective cohort study that enrolled 560 consecutive patients with ischemic cardiomyopathy. Patients were recruited from the heart failure outpatient clinic of Hippokration University Hospital, Athens, and were followed prospectively for a median duration of 43 months (IQR, 29–56). Patients were included in the study if they had a history of CAD and had a history of at least one hospital admission for heart failure. CAD was diagnosed based on a history of myocardial infarction or the presence of at least one major epicardial coronary artery stenosis of ≥50%, confirmed by invasive coronary angiography and accompanied by evidence of myocardial ischemia. Patients needed to be clinically stable for at least six months and receive optimal medical therapy. Heart failure was classified into HFrEF, HFmrEF, and HFpEF based on contemporary European Society of Cardiology guidelines.

Exclusion criteria included significant valvular or congenital heart disease, uncontrolled hypertension, acute decompensated heart failure, advanced renal impairment (eGFR < 30 mL/min/1.73 m^2^), advanced liver disease, chronic pulmonary disease, systemic inflammatory or malignant conditions, thyroid disorders, and previous transplantation. Written informed consent was obtained from all participants prior to inclusion.

Baseline demographic, clinical, and laboratory data were collected, including age, sex, BMI, presence of arterial hypertension, diabetes mellitus, dyslipidemia, and smoking history. Smoking status was classified as current (smoking within the past year), ex-smoker (ceased >1 year ago), or never smoker.

Follow-up was conducted through clinic or telephone visits by physicians to monitor the occurrence of major adverse cardiovascular events (MACE), defined as a composite of death, myocardial infarction, coronary revascularization, progression of coronary artery disease based on angiographic findings requiring adjustment of medical treatment, hospitalization for heart failure, stroke, and hospitalization for other cardiovascular causes, including systemic embolism or peripheral artery disease revascularization.

### 2.2. Assessment of Endothelial Function

Endothelial function was evaluated by assessing FMD of the right brachial artery, following fasting of at least 12 h and temporary withdrawal of vasoactive medications, as previously described in the literature [17]. Measurements were conducted in a temperature-controlled, quiet environment after a 10-min rest. Ultrasound imaging employed a Vivid e-ultrasound system (General Electric, Boston, MA, USA) with a 5.0–13.0 MHz linear transducer positioned 5 cm above the antecubital fossa. A pneumatic cuff distal to the probe was inflated to suprasystolic pressure for 5 min to induce reactive hyperemia. Post-cuff release, arterial diameter was manually measured with electronic calipers every 15 s for 2 min. FMD was expressed as the percentage increase from baseline diameter to peak dilation. All measurements were performed by a single experienced operator, with a blinded second observer verifying values to minimize bias.

### 2.3. Assessment of Arterial Stiffness

cf-PWV was utilized as a measure of arterial stiffness. Pulse transit time was recorded between the carotid and femoral arteries using a validated non-invasive device (SphygmoCor; AtCor Medical, NSW, Australia). The travel distance was measured as the difference between the distance from the suprasternal notch to the femoral artery and that from the carotid artery to the suprasternal notch [18]. PWV was computed as distance in meters divided by transit time in seconds (Supplementary Figure S1).

### 2.4. Echocardiographic Evaluation

Comprehensive transthoracic echocardiography was performed using a Vivid e-cardiovascular ultrasound system (General Electric, Boston, MA, USA) equipped with a 2.0–3.6 MHz phased array transducer. Left ventricular ejection fraction (LVEF) was quantified by the Simpson biplane method following established guidelines from international echocardiography societies [19].

### 2.5. Bioethics

All participants were informed about the study’s objectives and procedures, and written informed consent was obtained prior to enrollment. The study was conducted in accordance with the 1989 Declaration of Helsinki and received a priori approval from the Scientific Research Institute of Hippokration University Hospital.

### 2.6. Statistical Analysis

All variables were assessed for normality using probability-probability (P-P) plots. Variables with a normal distribution are presented as means ± standard deviation, while non-normally distributed variables are reported as median with interquartile range. Categorical variables are expressed as valid percentages within the respective subpopulations. For comparisons between two groups with normally distributed continuous variables, the *t*-test was employed and for non-normally distributed continuous variables Mann-Whitney test was performed. Differences in left ventricular ejection fraction (LVEF) between BMI categories were analyzed using one-way ANOVA or the Kruskal–Wallis test as appropriate. To account for multiple comparisons among the three groups, Bonferroni correction was applied. The chi-square test was used to examine differences in categorical variables. To explore the effect of variables on FMD or PWV, linear regression analysis was per-formed while adjusting for possible confounders. Cubic spline curves with four knots were used to model and visualize potential non-linear relationships between FMD and BMI and hazard ratio for the incidence of MACE.

The prognostic value of variables for major adverse cardiovascular events (MACE) was initially assessed using univariable Cox proportional hazards models. Variables with a *p*-value ≤ 0.10 or those deemed clinically important were subsequently included in multivariable Cox regression analyses. All *p*-values reported are from two-sided tests, with statistical significance defined as *p* < 0.05. To assess potential multicollinearity among predictors in the regression models, variance inflation factors (VIF) were calculated for all included variables, with a VIF threshold of 5 used to indicate significant collinearity. After calculating the variance inflation factors, no significant multicollinearity was detected among the variables examined. Sensitivity analyses were performed using Log-Rank tests to compare survival differences between obese and normal-weight patients, obese and non-obese patients, as well as between patients with FMD values above and below the mean, to evaluate the robustness of the primary findings, and are shown as Kaplan–Meier survival curves. Statistical analyses were conducted using SPSS version 26.0 (IBM SPSS Statistics, Armonk, NY, USA) and R version 4.4.2 (R Core Team, 2021).

## 3. Results

### 3.1. Baseline Characteristics

A total of 560 consecutive patients (89.6% male) with ischemic cardiomyopathy were prospectively followed for a median of 43 months (IQR, 29–56). The mean age was 63 ± 11 years. Based on BMI, 54.8% were overweight, 20.0% had class I obesity, 4.0% class II obesity, and 0.2% class III obesity. No patients were underweight. At study entry, 20.2% of patients had all three cardiometabolic risk factors (CMRFs: arterial hypertension, dyslipidemia, diabetes mellitus), whereas 9.1% had none. Based on LVEF, 55.4% had HFpEF, 50.9% had HFmrEF, and 23.8% had HFrEF. The mean FMD value was 4.92 ± 2.29%, and the mean cf-PWV was 8.99 ± 2.49 m/s. Baseline demographic and clinical characteristics are summarized in Table 1.

### 3.2. Comparisons Between BMI Categories

Patients were stratified into three BMI groups: normal weight (18.5–24.9 kg/m^2^; n = 114), overweight (25.0–29.9 kg/m^2^; n = 298), and obese (≥30.0 kg/m^2^; n = 132). Patients with obesity were significantly younger than those with normal weight (P_ANOVA_ = 0.03; post hoc *p* = 0.02), while no age differences were observed between the other groups (*p* = NS for all other comparisons). A positive family history was more frequent in obese patients compared with normal weight and overweight patients (34.8% vs. 26.3% vs. 23.5%, respectively; *p* = 0.04).

History of tobacco use was more prevalent among overweight patients compared with normal weight or obese individuals (84.6% vs. 73.7% vs. 78.8%, respectively; *p* = 0.03), mainly due to a higher proportion of ex-smokers (64.4% in overweight vs. 50.8% in both normal weight and obese groups; *p* < 0.01). The prevalence of all three CMRFs was similar across BMI categories (*p* = 0.63). Notably, FMD and cf-PWV values did not differ significantly between BMI groups. No additional differences in demographic or clinical characteristics across BMI categories were observed (Table 2).

### 3.3. Comparisons Based on Burden of Cardiometabolic Risk Factors

Further analysis compared patients with 0–2 CMRFs (n = 447) to those with all three CMRFs (n = 113). Patients with 3 CMRFs were significantly older (66 ± 10 vs. 62 ± 11 years; *p* ≤ 0.01). HFmrEF was more common among patients with 3 CMRFs (30.1% vs. 18.6%; *p* = 0.01), whereas the prevalence of HFrEF was comparable between the two groups. Interestingly, cf-PWV values were higher in patients with 3 CMRFs compared to those with 0–2 CMRFs (9.77 ± 2.97 vs. 8.78 ± 2.30 m/s; *p* ≤ 0.01). Conversely, no significant differences were observed in FMD values between the two groups (Table 3). Comparisons based on the simultaneous presence of all four cardiometabolic factors (obesity, diabetes mellitus, arterial hypertension, and dyslipidemia) were omitted due to small subgroup size (n = 30).

### 3.4. Arterial Function, Weight Status and Burden of Cardiometabolic Risk Factors

**Figure 1:**
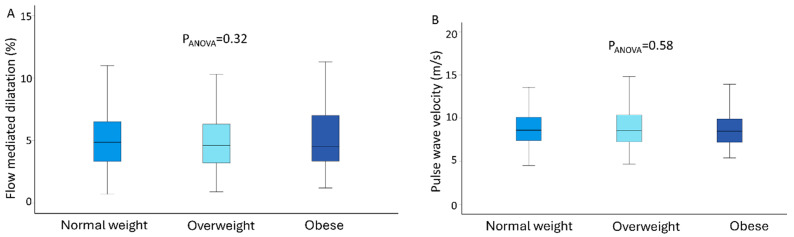
Box plots of arterial function parameters (arterial stiffness and endothelial function) according to weight status groups; (**A**). Flow-mediated dilation (FMD) values according to weight status groups; (**B**). Pulse wave velocity (PWV) values according to weight status groups.

FMD did not differ between normal weight, overweight and obese patients (5.18 ± 2.75 vs. 4.80 ± 2.13 vs. 5.00 ± 2.24%, respectively; P_ANOVA_ = 0.32). Additionally, no differences were observed regarding cf-PWV values between the 3 weight status strata (9.09 ± 2.85 m/s for normal weight vs. 8.74 ± 2.28 m/s for overweight vs. 8.99 ± 2.49 m/s for obese subjects; P_ANOVA_ = 0.58), as shown in Figure 1. A linear regression analysis with adjustment for various variables revealed no relationship between weight status or burden of CMRFs and FMD (Table 4).

**Figure 2:**
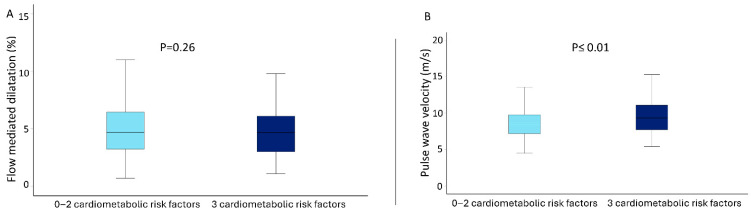
Box plots of arterial function parameters (arterial stiffness and endothelial function) according to the presence of 0–2 or 3 cardiometabolic risk factors; (**A**). Flow-mediated dilation (FMD) values according to clustering of cardiometabolic risk factors; (**B**). Pulse wave velocity (PWV) values according to clustering of cardiometabolic risk factors.

Age was a significant independent predictor for cf-PWV [beta = 0.10 (0.08–0.13); *p* < 0.001] after adjusting for multiple confounders. Furthermore, increasing burden of CMRFs was also associated with higher cf-PWV [beta = 0.39 (0.08–0.70); *p* = 0.01] (Table 4). To add to that, patients with 3 CMRFs had significantly increased cf-PWV in comparison to those with 0–2 CMRFs (9.77 ± 2.97 vs. 8.78 ± 2.30 m/s; *p* ≤ 0.01), as demonstrated in Figure 2. Linear regression analysis did not demonstrate other significant predictors for FMD or cf-PWV (Table 4).

### 3.5. Outcomes

The median follow-up was 43 months (IQR, 29–56). During this period, 39 patients died, 33 sustained a myocardial infarction, and 28 additionally required coronary revascularization according to standard clinical indications (8 received coronary artery bypass grafting, 20 had a percutaneous coronary intervention). Progression of CAD confirmed by coronary angiography and subsequently managed with optimal medical therapy occurred in 37 patients. In addition, 45 patients were hospitalized for heart failure, 13 experienced a stroke, and 22 were admitted for other cardiovascular causes. In total, 203 patients (36.3%) experienced a first major adverse cardiovascular event (MACE).

In the univariable proportional hazards analysis, the presence of all three CMRFs was predictive of a higher MACE risk [HR 1.42 (95% CI: 1.03–1.95); *p* = 0.03], as were diabetes mellitus [HR 1.40 (95% CI: 1.04–1.89); *p* = 0.03] and elevated creatinine levels [HR 1.02 (95% CI: 1.01–1.04); *p* = 0.03]. By contrast, obesity correlated with a 41% lower risk of MACE [HR 0.59 (95% CI: 0.38–0.92); *p* = 0.02]. Likewise, each 1% increment in FMD corresponded to an 8% lower risk [HR 0.92 (95% CI: 0.86–0.98); *p* = 0.01]. cf-PWV did not demonstrate prognostic value for incident MACE (Table 5).

**Figure 3:**
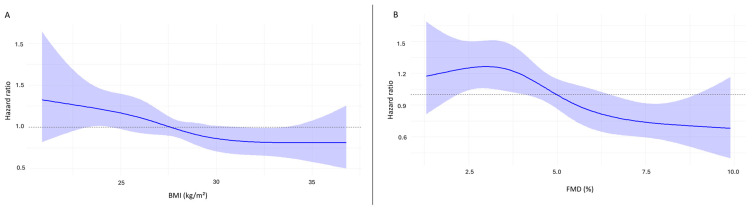
Cubic spline curve analyses illustrating the relationship between BMI and FMD and MACE risk. (**A**): Hazard ratio for MACE across BMI values, indicating lower risk for obese patients. (**B**): Association between flow-mediated dilation and MACE risk, demonstrating that lower FMD values correspond to progressively higher risk, while higher FMD values are linked to reduced risk. MACE: major cardiovascular adverse event; BMI: body mass index; FMD: flow-mediated dilation.

A cubic spline curve analysis showed that lower FMD values correlated with a progressively greater MACE risk, whereas higher FMD values were associated with lower risk (Figure 3). Similarly, the spline curve for BMI suggested that a value around 32.5 kg/m^2^ drives the favorable effect of obesity on MACE incidence, while values around 22.5 kg/m^2^ may have an unfavorable impact (Figure 3).

**Figure 4:**
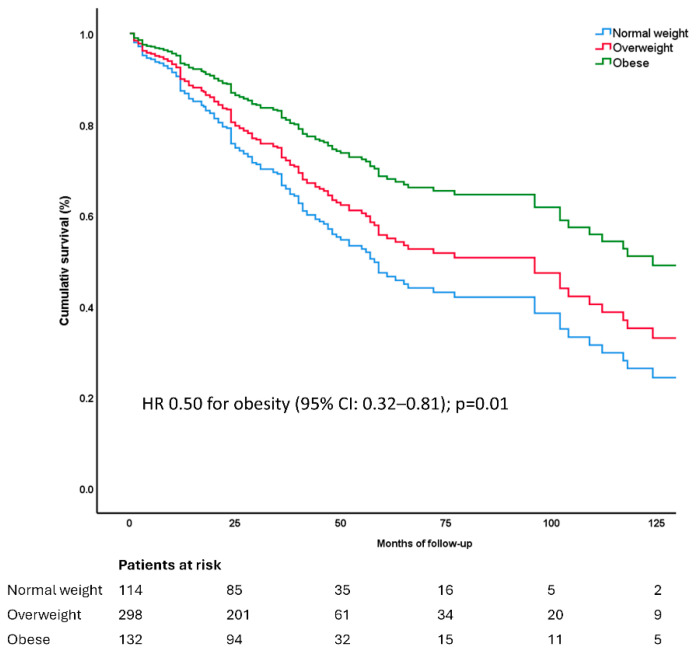
Survival curves from the multivariable proportional hazards model illustrating the survival according to weight status with obesity exerting significantly lower MACE risk compared to non-obese patients [HR 0.50 (95% CI: 0.32–0.81); *p* = 0.01], after adjustment for age, male gender, diabetes mellitus, history of CABG, CAD severity, flow-mediated dilation, creatinine and clustering of all 3 cardiometabolic risk factors (for abbreviations see Table 6).

In the multivariable proportional hazards model, both FMD [HR 0.93 (95% CI: 0.87–0.99); *p* = 0.03] and obesity [HR 0.50 (95% CI: 0.32–0.81); *p* = 0.01] remained independent protective predictors of MACE after adjustment for potential confounders (Table 6, Figure 4).

Sensitivity analyses using survival analysis showed a significantly better prognosis for obese patients compared to those of normal weight (Log-Rank test, *p* = 0.02) and compared to non-obese patients (*p* = 0.03). Furthermore, participants with flow-mediated dilation (FMD) values below the mean (FMD < 4.92%) had a worse prognosis than those with higher FMD values (Log-Rank test, *p* = 0.02) (Supplementary Figures S2–S4).

## 4. Discussion

The current prospective observational study of patients with ischemic cardiomyopathy evaluated the impact of CMRFs, including obesity, diabetes mellitus, arterial hypertension, and hyperlipidemia, on vascular function biomarkers (FMD and cf-PWV) and cardiovascular outcomes. The main findings were: (1) obesity was independently associated with favorable outcomes regarding incident MACE, (2) there was no difference regarding FMD and cf-PWV between weight status groups, (3) the clustering of all three main CMRFs (arterial hypertension, dyslipidemia, diabetes mellitus) was associated with unfavorable outcomes in univariable analysis and was reflected in impaired arterial wall properties, (4) higher FMD, a marker of endothelial function, exerted favorable predicting capacity, while cf-PWV, a surrogate for arterial stiffness, did not demonstrate prognostic significance.

### 4.1. Obesity and Vascular Function in Ischemic Cardiomyopathy

The current study shows a robust association between weight status and cardiovascular outcomes—specifically, a 50% reduction in the risk of MACE in obese compared to normal-weight patients, even after multivariable adjustment. This is consistent with extensive data in the heart failure population suggesting that being overweight or obese, while traditionally considered a major risk factor for cardiovascular disease or heart failure [20,21,22], does not always translate into poorer outcomes once the disease is established [23,24,25,26]. Multiple studies have demonstrated that in the context of heart failure, mildly to moderately obese patients, including those with reduced or preserved ejection fraction, show lower mortality and experience fewer cardiovascular events than normal-weight patients, which is referred to as obesity paradox in heart failure [8,9,10]. Particularly, underweight patients with heart failure demonstrate unfavorable cardiovascular outcomes [27,28]. Several hypotheses have been proposed, including increased metabolic reserves, protective adipokine profiles, and altered neurohormonal activation in obese patients [29,30] or reduced muscle mass as in sarcopenia, unintentional weight loss or cachexia reflecting more severe disease [31]. In addition, data from the Swedish heart failure registry show that obese patients can tolerate combined pharmacotherapy better, leading to achieving higher rates of optimal medical treatment compared to non-obese individuals [32].

FMD and cf-PWV are well-established biomarkers in cardiovascular disease, associated with the development of new-onset heart failure [11,12] as well as short- and long-term outcomes [14,15]. Although extensive data demonstrate that obese individuals generally exhibit impaired endothelial function and increased arterial stiffness prior to developing heart failure [11,33], evidence specifically examining the relationship between FMD and obesity in the context of ischemic cardiomyopathy remains scarce. In the present analysis, neither endothelial function (as measured by FMD) nor arterial stiffness (as measured by cf-PWV) differed between obese, overweight and normal-weight patients with ischemic cardiomyopathy. Furthermore, weight status did not predict FMD or cf-PWV values after adjustment for confounders in multivariable linear regression models. The underlying mechanism for these observations is unclear. It is plausible that endothelial dysfunction and arterial stiffness promote heart failure progression independently of weight status. Recent literature points to the role of adiponectin, a key adipokine secreted by adipose tissue, which may underlie the lack of difference in vascular function biomarkers between obese and non-obese subjects with ischemic heart failure. Adiponectin exerts protective effects by enhancing nitric oxide production, reducing vascular inflammation, and mitigating ceramide-induced damage. Higher adiponectin levels are linked to improved flow-mediated dilation and vascular health [34,35]; however, in chronic heart failure, strongly elevated levels may indicate resistance to adiponectin signaling and are associated with worse outcomes [34,36]. Therefore, in the context of ischemic cardiomyopathy, the interplay between obesity and vascular function is intricate and may be mediated by complex hormonal signaling.

Our findings demonstrate a significant association between obesity and lower MACE risk in ischemic cardiomyopathy. However, attributing an independent protective effect should be done cautiously, since residual confounding by other factors such as fitness status, muscle mass, and medications may influence outcomes. Furthermore, alternative adiposity markers like waist-to-hip ratio or visceral fat likely may provide greater prognostic accuracy than BMI alone and should be considered in future research. Large registry data also suggest that comorbidities and guideline-directed therapy influence prognosis more than infarct type itself (ST- vs. Non-ST-Elevation Myocardial Infarction), emphasizing the necessity of thorough risk assessment and optimized management for ischemic patients [37].

### 4.2. Clustering of Cardiometabolic Risk Factors

Patients who demonstrate higher cardiometabolic burden carry an increased risk due to this clustering of dysmetabolism, particularly in the context of heart failure [5,6,7]. The clustering of CMRFs—specifically arterial hypertension, dyslipidemia, and diabetes mellitus—predicted worse clinical outcomes in univariable analysis, emphasizing the additive risk associated with multiple coexisting dysmetabolic risk factors. This was also reflected by impaired vascular function, such as increased arterial stiffness, further underscoring the deleterious impact of combined CMRFs on the cardiovascular system.

Evidence from a pooled analysis of four large observational studies on 516,537 US patients shows that the clustering of obesity, diabetes mellitus and hypertension is associated with significantly worse outcomes regarding new-onset heart failure and heart-failure-related morbidity [5]. Patients with a high cardiometabolic burden should therefore be identified early and treated aggressively with optimal medical therapy, as strongly recommended by current clinical guidelines [3,4]. Intensive management of these interrelated risk factors is crucial to depress vascular damage, heart failure progression, and ultimately reduce the incidence of adverse cardiovascular events.

### 4.3. Prognostic Value of Flow-Mediated Dilation and Pulse Wave Velocity

The findings of the current study reinforce the strong and independent prognostic value of FMD in heart failure patients [14], particularly in those with ischemic heart failure [38]. Furthermore, FMD demonstrated a protective effect on the incidence of major cardiovascular events, showing a 7% reduction in MACE per 1% increase of FMD even after adjustment for multiple confounders. This highlights the value of FMD as a biomarker for risk stratification in ischemic cardiomyopathy and underscores the importance of targeting endothelial function to prevent adverse outcomes in this patient population. Conversely, cf-PWV, a marker of arterial stiffness, did not exhibit significant predictive capacity for MACE incidence, consistent with findings from other observational studies [14,16]. Other investigators have reported a significant prognostic value of PWV for predicting outcomes such as all-cause mortality, cardiovascular death, hospital readmissions, or their combination [11], illustrating a discrepancy in the current evidence regarding the role of PWV in risk stratification and prognostication in ischemic cardiomyopathy.

### 4.4. Limitations

This study has several limitations. The cohort consisted predominantly of male patients (89.6%) and patients with ischemic cardiomyopathy, which may limit the generalizability of the findings. The modest sample size and the low prevalence of patients with class II and III obesity could have reduced the power to detect subtle differences in vascular function biomarkers across BMI categories. The small sample size of patients presenting both obesity and all three cardiometabolic risk factors precluded a reliable analysis in this subgroup. Despite using multivariable adjustment models, residual confounding by unmeasured variables, such as medication adherence, lifestyle factors, or genetic predispositions, cannot be excluded. Nevertheless, the comprehensive phenotyping and sufficient median follow-up time of 43 months strengthen the clinical relevance of the results. A careful assessment of nutritional and inflammatory status in patients with ischemic heart disease is of fundamental importance, also using easily applicable derived indices. Although BMI has limitations and may be less accurate than other markers that account for fat distribution, such as waist-to-height ratio, particularly in heart failure patients [39], BMI remains a practical and widely used marker due to its simplicity and ease of calculation in clinical settings. When combined with novel indices like the Advanced Lung Cancer Inflammation Index (ALI), which integrates albumin and neutrophil-to-lymphocyte ratio, assessment of patient risk can be substantially enhanced without the need for complex or costly tools [40]. Additionally, while FMD and cf-PWV are established biomarkers in cardiovascular disease, they represent only specific aspects of vascular function, and other mechanisms influencing outcomes were not investigated.

Taken together, our findings lend further support to the concept that the obesity paradox in ischemic cardiomyopathy may not be mediated by improved vascular function as measured by either FMD or PWV. Rather, there may be other biological pathways—possibly related to metabolic reserves, systemic inflammation, neurohormonal activation through adiponectin, or medication pharmacokinetics in obese patients—that modulate risk and warrant further research.

## 5. Conclusions

Higher BMI and FMD each independently predict improved outcomes in ischemic cardiomyopathy, suggesting that the protective effect of obesity is unlikely to be mediated by vascular function markers. The clustering of cardiometabolic risk factors is a strong predictor of adverse events, underscoring the importance of comprehensive management of these risk factors.

## Figures and Tables

**Table 1:** Basic demographic and clinical characteristics.

Variable	Data
Age (years)	63 ± 11
Male gender—n/N (%)	502/560 (89.6%)
Body mass index (kg/m^2^)	27.96 ± 3.76
Normal weight: 18.5–24.99 kg/m^2^—n/N (%)	114/544 (21%)
Overweight: 25–29.99 kg/m^2^—n/N (%)	298/544 (54.8%)
Obesity—n/N (%)	132/544 (24.2%)
Category I: 30–34.99 kg/m^2^—n/N (%)	108/544 (20.0%)
Category II: 35–39.99 kg/m^2^—n/N (%)	22/544 (4.0%)
Category III: ≥40 kg/m^2^—n/N (%)	2/544 (0.2%)
Arterial hypertension—n/N (%)	421/560 (75.2%)
Diabetes mellitus—n/N (%)	153/560 (27.3%)
Dyslipidemia—n/N (%)	427/560 (76.3%)
Cardiometabolic risk factor burden	
No cardiometabolic risk factors—n/N (%)	51/560 (9.1%)
1 cardiometabolic risk factor—n/N (%)	130/560 (23.2%)
2 cardiometabolic risk factors—n/N (%)	266/560 (47.5%)
3 cardiometabolic risk factors—n/N (%)	113/560 (20.2%)
3 cardiometabolic risk factors and obesity—n/N (%)	30/544 (5.5%)
History of tobacco use—n/N (%)	453/560 (80.9%)
Currents smokers—n/N (%)	127/560 (22.7%)
Ex-smokers—n/N (%)	326/560 (58.2%)
Family history of coronary artery disease	153/560 (27.3%)
Previous myocardial infarction	264/560 (47.1%)
Previous CABG	85/560 (15.2%)
≥2 vessel disease—n/N (%)	196/530 (37%)
Atrial fibrillation	40/560 (7.1%)
Flow-mediated dilation (%)	4.92 ± 2.29
Carotid–femoral pulse wave velocity (m/s)	8.99 ± 2.49
Creatinine (mg/dL)	1.02 ± 0.37
LVEF (%)	50 (43–55)
Heart failure type	
HFrEF—n/N (%)	133/560 (23.8%)
HFmrEF—n/N (%)	117/560 (20.9%)
HFpEF—n/N (%)	310/560 (55.4%)

CABG: coronary artery bypass graft; LVEF: left ventricular ejection fraction; HFrEF: heart failure with reduced ejection fraction; HFmrEF: heart failure with mildly reduced ejection fraction; HFpEF: heart failure with preserved ejection fraction.

**Table 2:** Comparisons based on BMI categories.

Variable	Normal Weight (n = 114)	Overweight (n = 298)	Obesity (n = 132)	*p*
Age (years)	65 ± 12	63 ± 11	61 ± 10	0.03 * (ANOVA)
Male gender—n/N (%)	101/114 (88.6%)	274/298 (91.9%)	114/132 (86.4%)	0.18 (chi-square)
Body mass index (kg/m^2^)	23.33 ± 1.56	27.51 ± 1.34	32.97 ± 2.85	0.01 * (ANOVA)
Arterial hypertension—n/N (%)	79/114 (69.3%)	225/298 (75.5%)	106/132 (80.3%)	0.14 (chi-square)
Diabetes mellitus—n/N (%)	35/114 (30.7%)	80/298 (26.8%)	35/132 (26.5%)	0.70 (chi-square)
Dyslipidemia—n/N (%)	82/114 (71.9%)	223/298 (74.8%)	108/132 (81.8%)	0.16 (chi-square)
Presence of all 3 cardiometabolic factors—n/N (%)	24/114 (21.1%)	56/298 (18.8%)	30/132 (22.7%)	0.63 (chi-square)
History of tobacco use—n/N (%)	84/114 (73.7%)	252/298 (84.6%)	104/132 (78.8%)	0.03 (chi-square)
Currents smokers—n/N (%)	26/114 (22.8%)	60/298 (20.1%)	37/132 (28%)	0.20 (chi-square)
Ex-smokers—n/N (%)	58/114 (50.8%)	192/298 (64.4%)	67/132 (50.8%)	<0.01 (chi-square)
Family history of coronary artery disease	30/114 (26.3%)	70/298 (23.5%)	46/132 (34.8%)	0.04 (chi-square)
Previous myocardial infarction	53/114 (46.5%)	134/298 (45.0%)	60/132 (45.5%)	0.16 (chi-square)
Previous CABG	21/114 (18.4%)	45/298 (15.1%)	15/132 (11.4%)	0.30 (chi-square)
≥2 vessel disease—n/N (%)	47/110 (41.7%)	95/280 (33.9%)	47/125 (37.6%)	0.43 (chi-square)
Atrial fibrillation				
Flow-mediated dilation (%)	5.18 ± 2.75	4.80 ± 2.13	5.00 ± 2.24	0.32 (ANOVA)
Pulse wave velocity (m/s)	9.09 ± 2.85	8.74 ± 2.28	8.99 ± 2.49	0.58 (ANOVA)
Creatinine (mg/dL)	1.04 ± 0.35	0.99 ± 0.27	1.02 ± 0.37	0.49 (ANOVA)
LVEF (%)	48.5 (40–55)	50 (45–55)	50 (45–55)	0.42 (Kruskal–Wallis)
Heart failure type				0.14 (chi-square)
HFpEF—n/N (%)	57/114 (50.0%)	167/298 (56%)	78/132 (59.1%)	0.35
HFmrEF—n/N (%)	21/114 (18.4%)	61/298 (20.5%)	31/132 (23.5%)	0.61
HFrEF—n/N (%)	36/114 (31.6%)	70/298 (23.5%)	23/132 (17.4%)	0.03

* Significant statistical difference, *p* ≤ 0.05, between obesity and normal weight. CABG: coronary artery bypass graft; LVEF: left ventricular ejection fraction; HFrEF: heart failure with reduced ejection fraction; HFmrEF: heart failure with mildly reduced ejection fraction; HFpEF: heart failure with preserved ejection fraction.

**Table 3:** Comparisons based on the burden of cardiometabolic risk factors.

Variable	0–2 Cardiometabolic Risk Factors (n = 447)	3 Cardiometabolic Risk Factors (n = 113)	*p*
Age (years)	62 ± 11	66 ± 10	≤0.01 (*t*-test)
Male gender—n/N (%)	399/447 (89.3%)	103/113 (91.2%)	0.56 (chi-square)
Body mass index (kg/m^2^)	27.90 ± 3.72	28.20 ± 3.92	0.44 (*t*-test)
Arterial hypertension—n/N (%)	308/447 (68.9%)	113/113 (100.0%)	≤0.01 (chi-square)
Diabetes mellitus—n/N (%)	40/447 (8.9%)	113/113 (100.0%)	≤0.01 (chi-square)
Dyslipidemia—n/N (%)	314/447 (70.2%)	113/113 (100.0%)	≤0.01 (chi-square)
History of tobacco use—n/N (%)	358/447 (80.1%)	95/113 (84.1%)	0.34
Currents smokers—n/N (%)	107/447 (19.9%)	20/113 (17.7%)	
Ex-smokers—n/N (%)	251/447 (56.2%)	75/113 (66.4%)	
Family history of coronary artery disease	128/447 (28.6%)	25/113 (22.1%)	0.17 (chi-square)
Previous myocardial infarction	203/447 (45.4%)	60/113 (53.1%)	0.40 (chi-square)
Previous CABG	64/447 (14.3%)	21/113 (18.6%)	0.26 (chi-square)
≥2 vessel disease—n/N (%)	149/423 (35.2%)	47/107 (43.9%)	0.20 (chi-square)
Atrial fibrillation	32/447 (7.2%)	8/113 (7.1%)	1.00 (Fischer’s test)
Flow-mediated dilation (%)	4.98 ± 2.33	4.70 ± 2.11	0.26 (*t*-test)
Pulse wave velocity (m/s)	8.78 ± 2.30	9.77 ± 2.97	≤0.01 (*t*-test)
Creatinine (mg/dL)	1.00 ± 0.34	1.12 ± 0.43	0.01 (*t*-test)
LVEF (%)	50 (43–55)	47 (43–55)	0.36 (Mann–Whitney)
Heart failure type			0.02 (chi-square)
HFpEF—n/N (%)	257/447 (57.5%)	53/447 (46.9%)	<0.01
HFmrEF—n/N (%)	83/447 (18.6%)	34/113 (30.1%)	0.01
HFrEF—n/N (%)	107/447 (23.9%)	26/113 (23.0%)	0.93

CABG: Coronary artery bypass graft; LVEF: left ventricular ejection fraction; HFrEF: heart failure with reduced ejection fraction; HFmrEF: heart failure with mildly reduced ejection fraction; HFpEF: heart failure with preserved ejection fraction.

**Table 4:** Multivariable linear regression analysis for the association of predictors of flow-mediated dilation and pulse wave velocity.

	**Linear Regression Analysis for the Association of FMD (Dependent Variable) with Various Predictors**		
**Variable**	**b**	**95% CI**	***p* Value**
Age (per year)	−0.02	−0.04–0.01	0.23
Male gender *	−0.73	−1.53–0.08	0.08
History of CABG *	0.02	−0.73–0.76	0.96
History of myocardial infarction *	0.06	−0.34–0.46	0.78
Atrial fibrillation *	1.02	0.6–1.97	0.04
Pulse wave velocity (per m/s)	−0.02	−0.12–0.09	0.74
Creatinine (per mg/dL)	0.46	−0.31–0.22	0.24
Body mass index categories: reference category normal weight	−0.23	−0.57–0.12	0.19
Burden of cardiometabolic risk factors: reference category: none	−0.13	−0.69–0.43	0.66
Ejection Fraction (per %)	0.01	−0.02–0.03	0.76
Smoking History *	−0.43	−1.03–0.18	0.16
≥2 vessel CAD: reference group 1 vessel disease	0.12	−0.24–0.48	0.51
	**Linear Regression Analysis for the Association of PWV (Dependent Variable) with Various Predictors**		
**Variable**	**b**	**95% CI**	***p* Value**
Age (per year)	0.10	0.08–0.13	<0.001
Male gender *	0.45	−0.41–1.31	0.30
History of CABG *	−0.05	−0.84–0.75	0.91
History of myocardial infarction *	−0.24	−0.67–0.18	0.26
Atrial fibrillation *	0.29	−0.73–1.31	0.58
Flow-mediated dilation (per %)	−0.02	−0.14–0.10	0.72
Creatinine (per mg/dL)	0.21	−0.60–1.02	0.61
Body mass index categories: reference category normal weight	−0.02	−0.39–0.34	0.90
Burden of cardiometabolic risk factors: reference category: none	0.39	0.08–0.70	0.01
Ejection Fraction (per %)	0.01	−0.02–0.04	0.64
Smoking History *	0.19	−0.45–0.83	0.56
≥2 vessel CAD: reference group 1 vessel disease	0.28	−0.10–0.66	0.15

* Reference category: absence of characteristic. FMD: flow-mediated dilation; PWV: pulse wave velocity; CABG: coronary artery bypass graft; CAD: coronary artery disease.

**Table 5:** Univariable Cox Proportional Hazards for major adverse cardiovascular events.

Variable	HR	95% CI	*p* Value
Age (per year)	1.01	0.99–1.02	0.35
Male gender *	1.18	0.72–1.95	0.51
Body mass index (per kg/m^2^)	0.96	0.93–1.00	0.06
Body mass index categories: reference category normal weight			
Overweight (BMI 25–29.99 kg/m^2^)	0.88	0.63–1.23	0.47
Obesity (BMI ≥ 30 kg/m^2^)	0.59	0.38–0.92	0.02
Arterial Hypertension *	1.20	0.86–1.66	0.28
Diabetes mellitus *	1.40	1.04–1.89	0.03
Dyslipidemia *	1.03	0.74–1.44	0.85
Presence of all three cardiometabolic factors **	1.42	1.03–1.95	0.03
Presence of cardiometabolic factors: reference group non-cardiometabolic risk factors			
Presence of one cardiometabolic factor	0.98	0.56–1.72	0.94
Presence of two cardiometabolic factors	1.05	0.63–1.77	0.85
Presence of three cardiometabolic factors	1.45	0.84–2.52	0.18
History of tobacco use	1.00	0.69–1.44	1.00
Family history of coronary artery disease	1.07	0.79–1.46	0.66
Previous myocardial infarction	1.04	0.79–1.37	0.78
History of CABG	1.59	1.14–2.12	0.01
CAD Severity: reference group 1 vessel disease			
2-Vessel Disease	1.29	0.91–1.81	0.15
3-Vessel Disease	1.57	1.08–2.28	0.02
Atrial fibrillation *	0.74	0.40–1.36	0.34
Flow-mediated dilation (%)	0.92	0.86–0.98	0.01
Pulse wave velocity (m/s)	1.04	0.98–1.11	0.22
Creatinine (mg/dL)	1.02	1.01–1.04	0.03
LVEF category: reference group LVEF ≥ 50%	1.01	0.99–1.02	0.33
HFmrEF (LVEF 41–49%)	1.39	0.89–2.17	0.15
HFrEF (LVEF ≤ 40%)	1.34	0.88–2.06	0.17

* Reference category: absence of characteristic. ** Reference category: presence of none. one or two cardiometabolic factors. BMI: Body mass index; CABG: Coronary artery bypass graft; LVEF: left ventricular ejection fraction; HFrEF: heart failure with reduced ejection fraction; HFmrEF: heart failure with mildly reduced ejection fraction.

**Table 6:** Multivariable Cox Proportional Hazards for major adverse cardiovascular events.

Variable	HR	95% CI		*p* Value
Age (per year)	1.00	0.99–1.02		0.80
Male gender *	0.85	0.48–1.50		0.56
Body mass index categories: reference category normal weight				
Overweight (BMI 25–29.99 kg/m^2^)	0.78	0.55–1.12		0.19
Obesity (BMI ≥ 30 kg/m^2^)	0.50	0.32–0.81		0.01
Diabetes mellitus *	1.26	0.65–2.45		0.50
History of CABG	1.43	0.94–2.17		0.10
CAD Severity: reference group 1 vessel disease				
2-Vessel Disease	1.14	0.77–1.68		0.51
3-Vessel Disease	1.34	0.84–2.11		0.22
Flow-mediated dilation (%)	0.93	0.87–0.99		0.03
Creatinine (mg/dL)	0.72	0.41–1.25		0.24
Presence of all three cardiometabolic factors **	1.30	0.65–2.59		0.46

* Reference category: absence of characteristic. ** Reference category: presence of none. one or two cardiometabolic factors. BMI: Body mass index; CAD: coronary artery disease; CABG: Coronary artery bypass graft.

## Data Availability

The data of this study are available from the corresponding author upon reasonable request.

## Supplementary Materials

The following supporting information can be downloaded at: https://www.mdpi.com/article/10.3390/jcm14207397/s1, Figure S1: Non-invasive assessment of arterial stiffness by measuring carotid-femoral pulse wave velocity; Figure S2: Kaplan-Meier survival analysis between obese patients (BMI ≥ 30 kg/m^2^) and patients with normal weight (BMI 18.51–24.99 kg/m^2^); Figure S3: Kaplan-Meier survival analysis between obese patients (BMI ≥ 30 kg/m^2^) and non-obese patients (BMI 18.51–29.99 kg/m^2^); Figure S4: Kaplan–Meier survival analysis between participants with FMD values below the mean value (FMD < 4.92%) and patients with FMD values ≥ 4.92%.
