# Supplementary material for: Prognostic Impact of Obesity, Cardiometabolic Risk Factors, and Vascular Function Markers on Outcomes in Ischemic Cardiomyopathy
Source: J Clin Med. 2025 Oct 20;14(20):7397. doi: 10.3390/jcm14207397 (PMC12565017; doi:10.3390/jcm14207397)
Supplement: Supplementary file 1 [file jcm-14-07397-s001.zip › jcm-3877532-supplementary.pdf]

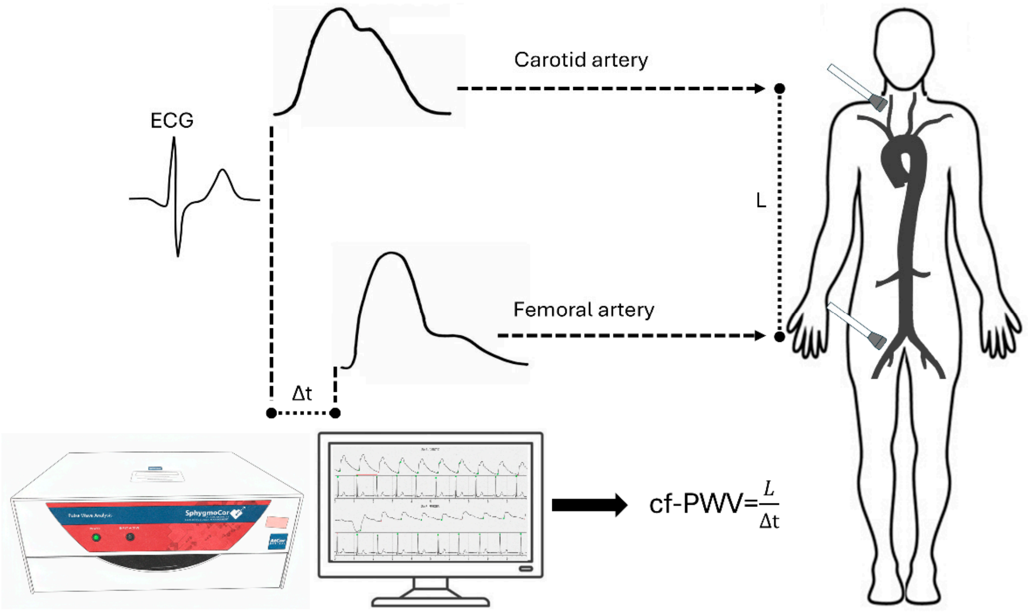

Supplementary Figure S1: Non-invasive assessment of arterial stiffness by measuring carotid-femoral pulse wave velocity. Pulse transit time was recorded between the carotid and femoral arteries using a validated non-invasive device (SphygmoCor; AtCor Medical). The travel distance was measured as the difference between the distance from the suprasternal notch to the femoral artery and that from the carotid artery to the suprasternal notch. Carotid-femoral pulse wave velocity (cf-PWV) was computed as distance (L) in meters divided by transit time in seconds (Δt).

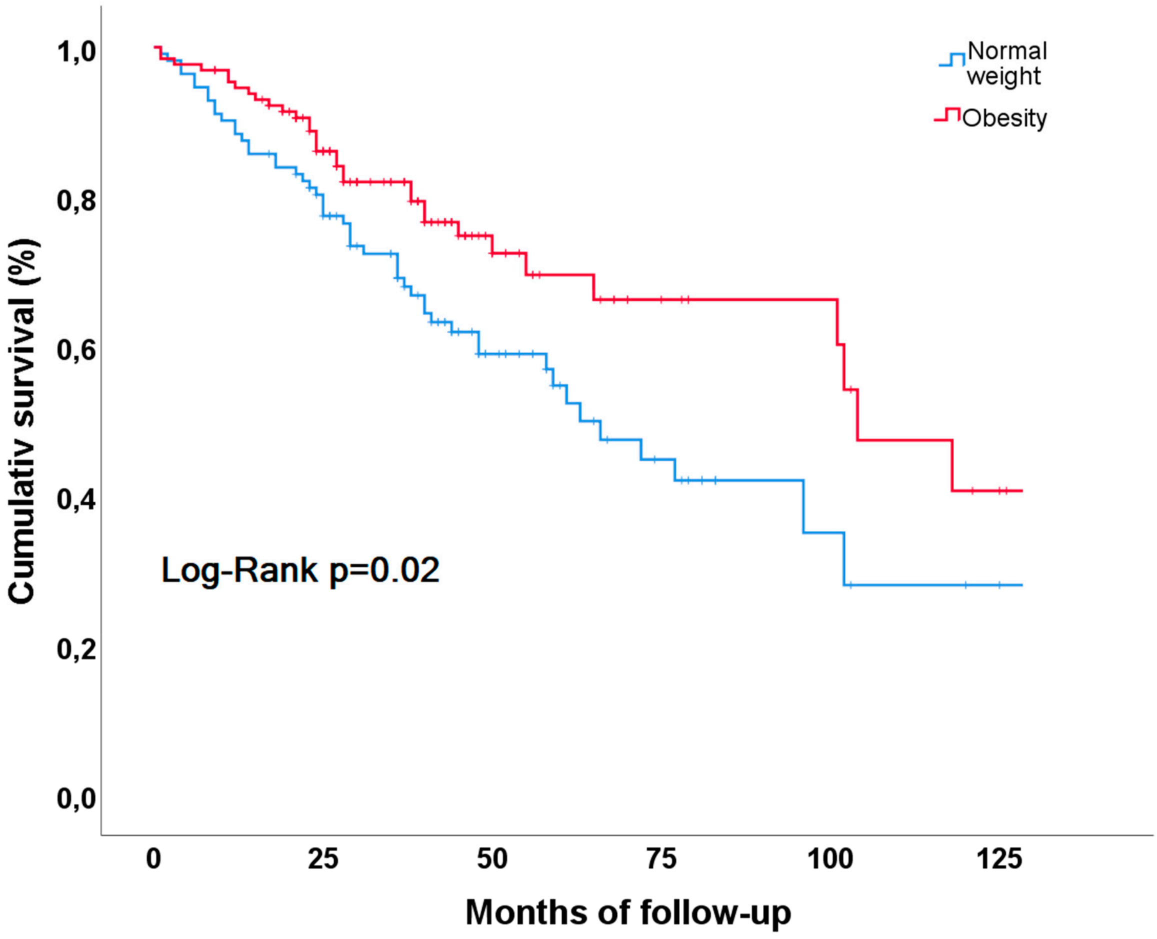

Supplementary Figure S2: Kaplan-Meier survival analysis between obese patients (BMI ≥30 kg/m^2) and patients with normal weight (BMI 18.51-24.99 kg/m^2) .

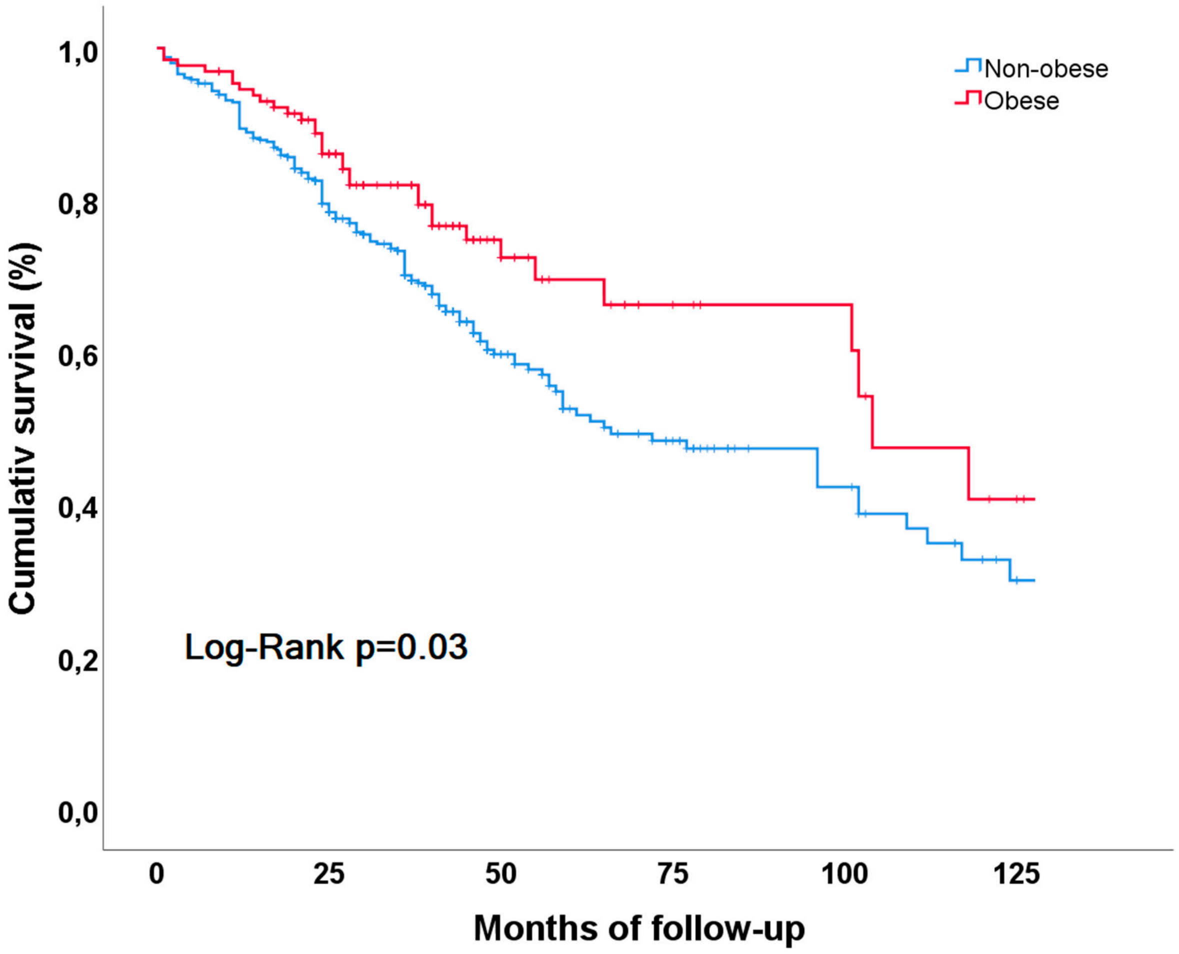

Supplementary Figure S3: Kaplan-Meier survival analysis between obese patients (BMI ≥30 kg/m^2) and non-obese patients (BMI 18.51-29.99 kg/m^2) .

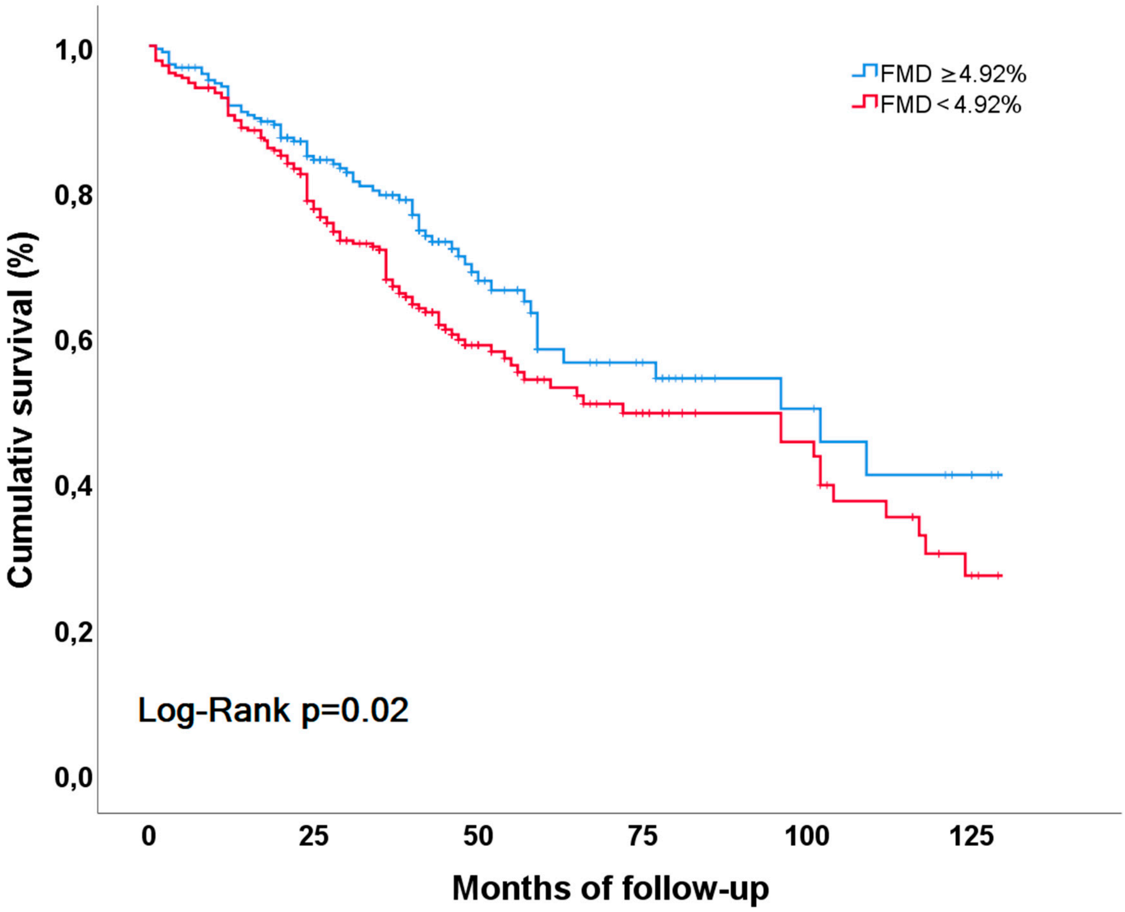

Supplementary Figure S4: Kaplan-Meier survival analysis between participants with FMD values below the mean value (FMD <4.92%) and patients with FMD values ≥4.92%. FMD: Flow-mediated dilatation
